# Sedation and Analgesia in Intensive Care: A Comparison of Fentanyl and Remifentanil

**DOI:** 10.1155/2011/650320

**Published:** 2011-07-02

**Authors:** F. Cevik, M. Celik, P. M. Clark, C. Macit

**Affiliations:** ^1^Medical Department, Abbott Pharmaceuticals, Meral Plaza, Umraniye, Istanbul, Turkey; ^2^ICU Department, Ministry of Health Teaching and Research Hospital, Göztepe, Istanbul, Turkey; ^3^Yeditepe University School of Pharmacy, 26 August Campus, 34755 Atasehir, Istanbul, Turkey

## Abstract

Optimal sedation and analgesia are of key importance in intensive care. The aim of this study was to assess the quality of sedoanalgesia and outcome parameters in regimens containing midazolam and either fentanyl or remifentanil. A prospective, randomized, open-label, controlled trial was carried out in the ICU unit of a large teaching hospital in Istanbul over a 9-month period. Thirty-four patients were randomly allocated to receive either a remifentanil-midazolam regimen (*R* group, *n* = 17) or a fentanyl-midazolam regimen (*F* group, *n* = 17). A strong correlation between Riker Sedation-Agitation Scale (SAS) and Ramsey Scale (RS) measurements was observed. Comparatively, remifentanil provided significantly more potent and rapid analgesia based on Behavioral-Physiological Scale (BPS) measurements and a statistically nonsignificantly shorter time to discharge. On the other hand, remifentanil also caused a significantly sharper fall in heart rate within the first six hours of treatment.

## 1. Introduction

 Virtually every patient admitted into ICU is administered analgesia and sedation therapy. Therefore, sedation and pain control are crucial in the management of intensive care unit (ICU) patients. Because of the multiplicity of patients admitted to ICU, it is difficult to define a standard procedure for ICU sedation. One may encounter a variety of pathologies of different grades of severity; in addition, the associated morbidity, the circulatory instability, and the pharmacodynamic alterations in critically ill patients can make treatment guideline difficult to establish and implement [1]. Precise control of the depth of sedation is often not well managed; patients are frequently over- or undersedated with an accompanying increase in morbidity, mortality, and economic cost. 

 The provision of effective analgesia and sedation for patients in ICU is important in controlling pain, dyspnea, and delirium; relieving agitation and anxiety; decreasing oxygen consumption; providing amnesia; preventing seizure; maintaining brain function; applying neuromuscular blockage; aiding compliance with mechanical ventilation; thereby maintaining patient comfort [2–4]. Agents such as propofol and midazolam are commonly used for sedation in ICU because of their effectiveness and relatively short elimination half-lives. The risk for accumulation and delayed recovery with these agents appears to be lower than that with traditional opioids. Consequently, opioid dose is usually minimized, with clinicians choosing to manipulate the sedative dose to maintain optimal patient comfort. On the other hand, analgesia-based sedation techniques, which focus on patient comfort rather than on patient sedation by catering to the analgesic needs of the patient and adding a sedative only if necessary, have become more established in the ICU setting. Both approaches currently have certain limitations [3], because when administered over several hours or even days, elimination of most drugs used for analgesia and sedation may be reduced in critically ill patients secondary to impairment in organ-dependent elimination. This can result in delayed emergence from sedation after discontinuation of administration, increased time on the ventilator and in ICU, and therefore increased costs. Moreover, prolonged sedation may not only have economic consequences but also medical ones, such as failure to recognize cerebral insult, immunosuppression, or venous stasis leading to thromboembolism [1]. A specialized ICU team, including physicians, nurses, and pharmacists, has important responsibilities in maximizing the clinical effect of medicines, minimising the risk of treatment-induced adverse events, and minimising the expenditures for pharmacological treatments. [5]. 

 In this study, we aimed to assess the quality of sedoanalgesia and outcome parameters in regimens containing either fentanyl or remifentanil.

## 2. Methods

### 2.1. Study Population and Study Center

 This study was a prospective, randomized, open-label, and controlled trial approved by the Research Ethics Committee of Ministry of Health Göztepe Research & Teaching Hospital in Istanbul, Turkey. This trial was conducted in the 16-bed capacity ICU of the above-mentioned hospital. This study was carried out over a period between September 1, 2007 and May 31, 2008. Thirty-four patients were included in the study, and the patients were randomly allocated to one of two groups. All of the patients were provided with mechanical ventilation by Simultaneously Intermediate Mechanical Ventilation/Volume Control (SIMV/VC) mode.

### 2.2. Eligibility Criteria

#### 2.2.1. Inclusion Criteria

Age ≥ 18; patient requiring mechanical ventilation and sedation.

#### 2.2.2. Exlusion Criteria

 Age ≤ 18; patients who have neuromuscular disease; patients who are receiving neuromuscular blockers; patients with a known or suspected allergy or intolerance to midazolam, fentanyl, or remifentanil; patients who die during the study period; patients who use toxic substances; alcoholic patients; patients suspected of being pregnant; patients who are moribund, that is, who are classified as ASA grade V according to the American Society of Anesthesiologists (not expected to live > 24 hours).

Because most patients were transferred to ICU from brain surgery theatre and were thus already sedated on admission, the WHO chronic pain was not reckoned to be applicable as an exclusion criterion. By the same token, given the patient group investigated in this study, confusion assessment was not deemed necessary.

### 2.3. Drugs and Infusion Regimens

 Remifentanil (5 mg), fentanyl (10 mg), and midazolam (60 mg) were diluted with 0.9% sodium chloride (150 mL). The concentration of remifentanil solution was 0.03 mg/mL, fentanyl solution 0.06 mg/mL, and midazolam solution 0.4 mg/mL. In total, 34 patients were randomized to receive either a remifentanil-midazolam regimen (*R* group, *n* = 17) or a fentanyl-midazolam regimen (*F* group, *n* = 17), for analgesia and sedation in the ICU. The *R* group received remifentanil at an initial dose of 0.05 *μ*g/kg/min, and the *F* group received fentanyl at an initial dose of 0.015 *μ*g/kg/min. Both groups received midazolam infusion at an initial dose of 0.03 mg/kg/h (see Figure 7). 

No adjuvant analgesics such as paracetamol or morphine were administered, and no rescue pain therapy was provided. Bolus doses of remifentanil, fentanyl, or midazolam were not allowed in the protocol and therefore were not administered.

### 2.4. Measurement Scales

In many government hospitals in Turkey, such as the center where the study was performed, there is no written protocol governing the use of agents providing sedoanalgesia; physicians tend to rely on their clinical experience and an assessment of the patient's hemodynamic status to determine doses. In this study, however, in order to maintain an adequate level of sedoanalgesia, an RS score of three or less (≤3) was reckoned to warrant an incremental increase in the opioid maintenance dose. In the *F* group an increase in fentanyl by 0.01 *μ*g/kg/min and in the *R* group an increase in remifentanil by 0.05 *μ*g/kg/min were administered (Figure 7). 

After the protocol was initiated, daily interruption of analgesia and sedation was performed. Once this daily interruption procedure was completed, the dosages of opioid analgesics and sedative were readjusted in the light of the patients' needs, as described above. This daily procedure was continued until analgesia-based sedation therapy was no longer required. GCS and APACHE II scores were used to assess level of consciousness of patients and also severity of their diseases. The use of GCS was judged appropriate because most patients were referred from the brain surgery service.

### 2.5. Measurements and Records

 The patients' demographic data and medical history, and also details of their physical examination, were recorded before starting sedation and analgesia therapy. APACHE II score and GCS were recorded daily in order to assess the seriousness of the patients' condition. Unless specified, the following parameters were recorded every two hours on the first day of admission and every six hours thereafter: scores of RS and SAS (in order to assess the quality of sedation); BPS score (to assess quality of analgesia); invasive BP, CVP, HR, body temperature (to assess hemodynamic status and organ dysfunction—performed hourly throughout the study); PaCO_2_, PaO_2_, SpO_2_, and pH (lung function assessment); ALT and AST (for hepatic function), BUN and SrCr (renal function measurement). The total daily doses of remifentanil, fentanyl and also midazolam were recorded daily. In addition, the time to tracheal extubation after sedation interruption, time to ICU discharge, and time of stay in ICU were recorded. Furthermore, adverse drug reactions and also the use of agents used to treat these side effects were documented.

### 2.6. Statistical Analysis

 All the variables are expressed as mean ± SD. Differences between groups were assessed using mixed-design ANOVA (one way for independent groups and repeated measures) with post hoc Scheffe's test. Correlation coefficients were calculated using Pearson correlation analysis. A value of *P* < 0.05 in a two-tailed distribution was considered statistically significant. All significant differences used in this paper were further subjected to power analysis.

## 3. Results

### 3.1. Demographics

 A total of 34 patients were enrolled in the study, of which 32 were able to be evaluated. Two patients (one patient in the fentanyl/midazolam group and one patient in the remifentanil/midazolam group) had to be excluded because they died before weaning during the study. The baseline characteristics of the 32 patients included in the study are summarized in Table 1. The small number of subjects and the heavy proportion of neurosurgical patients can be explained by the fact that the ICU was a referral centre for postoperative brain surgery patients such as those with spinal and brain tumours, intracerebral aneurysms, intracranial abscesses, and hematomas. In addition, during the study period the ICU was temporarily closed for some pressing repair work on the fabric of the unit. 

There were no significant differences between the groups with respect to baseline hemodynamic parameters and also demographic data (*P* > 0.05). 

### 3.2. Hemodynamics

**Figure 1:**
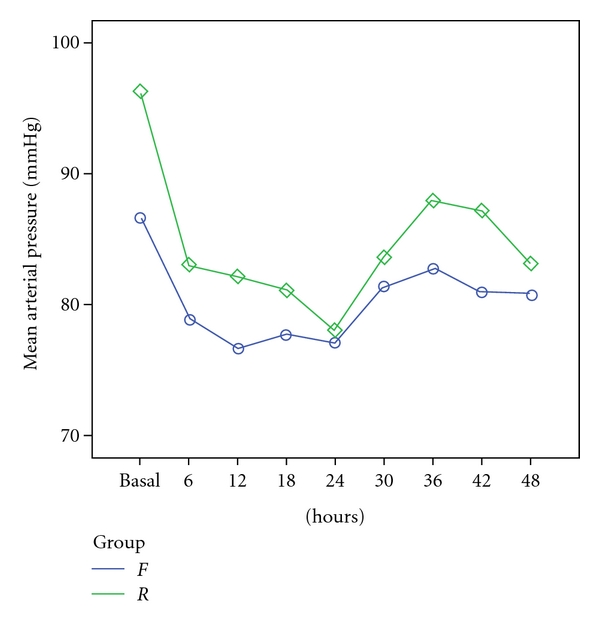
Mean arterial pressure (mmHg) in *F* and *R* groups before induction (basal) and during the first 48 hours of the sedation and analgesia therapy (*P* > 0.05) (mean ± SD).

 With respect to MAP, there was no significant differences observed between the *F* and *R* groups (*P* > 0.05). However there were significant variations in MAP within each group, during the course of time. For example, within both groups a significant fall in MAP was observed in the first six hours after sedation was initiated (*P* < 0.05). In addition, as can be seen in Figure 1, during this period the *R* group demonstrated a greater, but statistically nonsignificant, reduction in MAP than the *F* group. 

**Figure 2:**
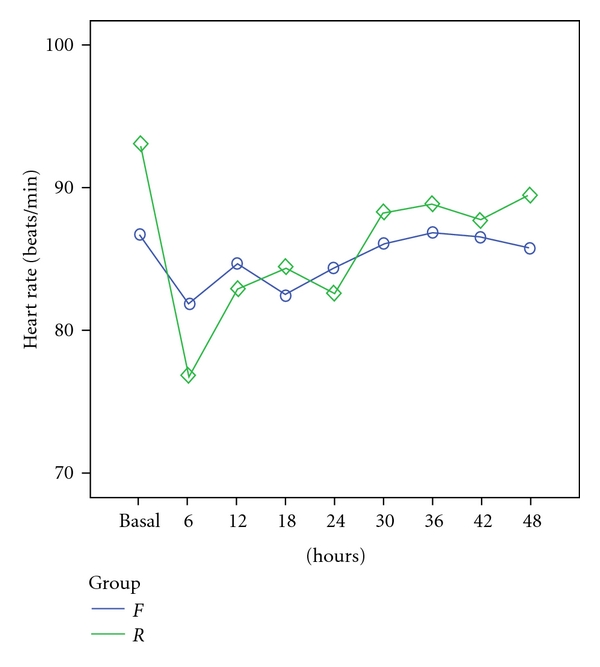
Mean heart rate (beats/min) in *F* and *R* groups before induction (basal) and during the first 48 hours of the sedation and analgesia therapy (*P* > 0.05).

 In regard to HR, there were no significant differences between the *F* and *R* groups at baseline (*P* > 0.05). However, in the first six hours after analgosedation was initiated, a significant difference in HR was observed between the groups (*P* < 0.05). During this time, as can be seen in Figure 2, remifentanil caused a sharp reduction in HR, while the HR of patients receiving fentanyl remained relatively stable. 

There was no significant differences between the *F* and *R* groups (*P* > 0.05) in terms of body temperature.

### 3.3. Analgesics and Sedative Dosage

**Figure 3:**
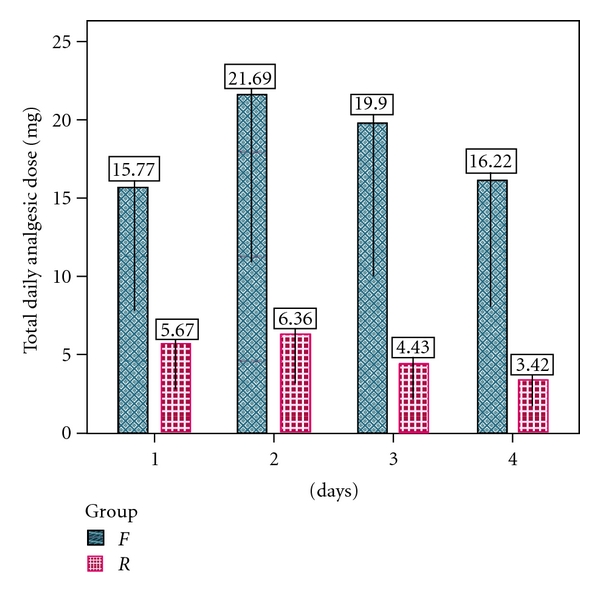
Total daily analgesic dosage (mg) of the patients in *F* and *R* groups on days 1, 2, 3, and 4 of the sedation and analgesia therapy (*P* > 0.05).

 Daily interruption was performed in order to provide controlled sedation and analgesia. In addition, doses of midazolam, fentanyl, and remifentanil were titrated according to patients' requirements and hemodynamics. The mean daily administered dosages of fentanyl and remifentanil were 18.41 ± 2.87 mg (range 0.015 to 0.03 *μ*g/kg/min) and 4.97 ± 1.31 mg (range 0.05 to 0.1 *μ*g/kg/min), respectively (see Figure 3). Only the first four days of sedoanalgesic treatment were recorded because after this the patients' sedoanalgesia was terminated in preparation for discharge (see Figure 6). If sedoanalgesia treatment lasted less than four days, no patient data for the period after stopping sedoanalgesia was recorded. 

**Figure 4:**
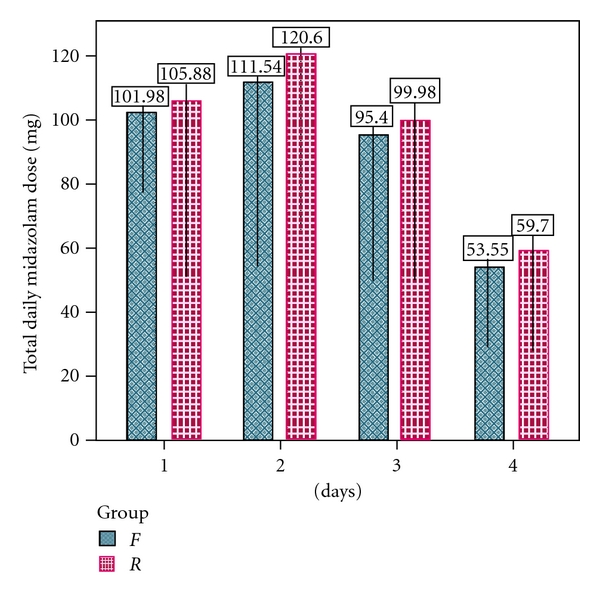
Total daily midazolam dose (mg) of patients in *F* and *R* groups on days 1, 2, 3, and 4 of the sedation and analgesia therapy (*P* > 0.05).

 The mean daily administered dosages of midazolam in the *F* group were 90.62 ± 25.58 mg (range 0.03 to 0.09 mg/kg/h) and in the *R* group were 96.54 ± 26.05 mg (range 0.03 to 0.12 mg/kg/h). With respect to analgesic dosages, there were no significant differences between the *F* and *R* groups (*P* > 0.05), but significant differences were found within the groups (*P* < 0.05) (see Figure 4). The midazolam requirements in the *F* group were lower than those in the *R* group. However, this finding was not statistically significant. 

### 3.4. Quality of Sedation and Analgesia

**Figure 5:**
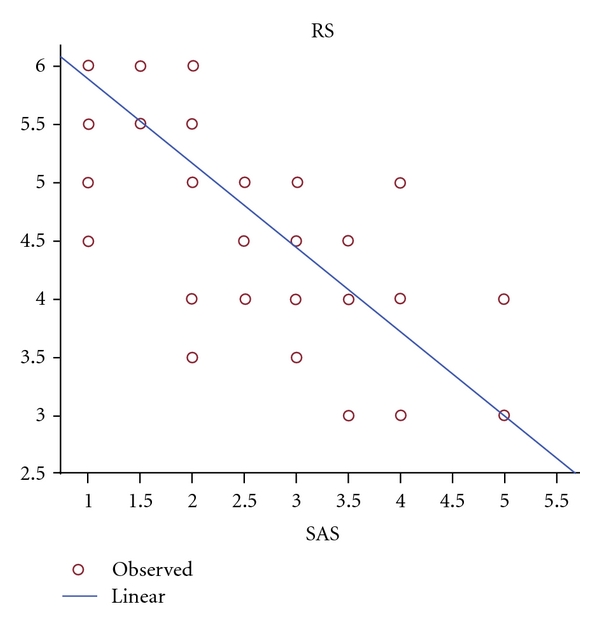
Correlation between RS and SAS in patients sedated with midazolam during the four days of sedoanalgesia (*R* = −0.822, *P* < 0.001).

 As can be observed in Table 2, at the induction of sedation, the mean RS for *F* and *R* groups was between 4 and 5, while the mean SAS for both groups was between 2 and 3. There were no significant differences between the groups (*P* > 0.05). On the other hand, there was a strong negative correlation between RS and SAS (*R* = 0.822, *P* < 0.001) (Figure 5).

At the induction of sedation (baseline), the mean BPS for *F* and *R* groups was between 3 and 4 which indicate “mild to moderate pain.” At baseline, there were no significant differences between the groups (*P* > 0.05). While in both groups the BPS scores resembled each other before opioid induction, after analgesic therapy was initiated a significant reduction was recorded compared with baseline values (*P* < 0.001). In comparison with the *F* group, the *R* group provided a significantly greater reduction in the first 10 minutes (*P* < 0.05).

**Figure 6:**
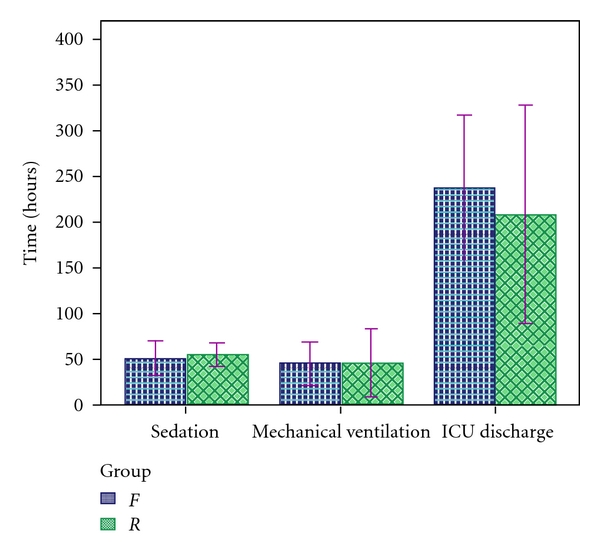
Time of sedation, mechanical ventilation, and ICU discharge (hours) in *F* and *R* groups.

**Figure 7:**
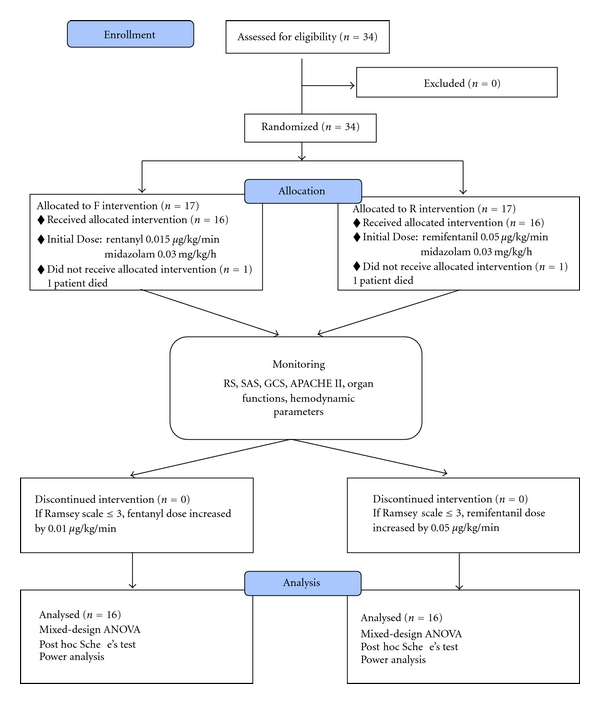
Study algorithm.

 The mean sedation times within *F* and *R* groups were 48.56 ± 32.7 and 54 ± 21.19 hours (*P* > 0.05), respectively. There were no significant differences within either group (*P* > 0.05) or between groups (Figure 6).

 The mean mechanical ventilation times of the *F* and *R* groups were 45.75 ± 47.13 and 45.75 ± 74.71 hours (*P* > 0.05). Regarding mechanical ventilation time, there were no statistically significant differences between both groups (*P* > 0.05) (see Figure 6). 

 The mean times to ICU discharge of *F* and *R* groups were 237 ± 159.89 hours and 208.69 ± 239.06 hours (*P* > 0.05), respectively. With regard to ICU discharge time, there were no significant differences between groups statistically (*P* > 0.05). However, as can be seen in Figure 6, there was a non-significant difference between groups, with the time until ICU discharge of the *R* group being shorter than that of the *F* group.

### 3.5. Organ Dysfunction

 With respect to arterial blood gas data, there were no significant differences between the *F* and *R* groups at 0, 24, 48, 72, or 96 hours (*P* > 0.05), but regarding PaO_2_, significant differences were found within both groups at 24 and 48 hours (*P* = 0.015 and *P* = 0.017, resp.) Concerning plasma pH, there were significant differences within both groups at 24 and 48 hours (*P* = 0.013 and *P* = 0.014, resp.). In addition, regarding SpO_2_, significant differences were found within both groups at time 0 and 24 hours (*P* = 0.016 and *P* = 0.018, resp.). With respect to PaCO_2_, there were no significant differences within the groups at time 0, 24, 48, 72, or 96 hours (*P* > 0.05).

 Regarding BUN, an examination of Table 3 shows that there were no significant differences between either group at 0, 24, or 48 hours (*P* > 0.05). However, significant differences were found between both groups at 72 and 96 hours (*P* = 0.014 and *P* = 0.021, resp.). The finding relating to the difference in BUN at 96 hours had a high power value of 0.991 according to power analysis. With respect to serum creatinine, significant differences were not observed either within or between the groups at 0, 24, 48, 72, and 96 hours (*P* > 0.05).

 As can be seen in Table 4, with respect to ALT, significant differences were not found between the groups at 72 or 96 hours (*P* > 0.05). However, there were significant differences between both groups at times of 0, 24, and 48 hours (*P* = 0.015, *P* = 0.029, and *P* = 0.021, resp.). Concerning AST, significant differences were not observed between the groups at 0, 24, 48, 72, or 96 hours (*P* > 0.05).

### 3.6. Adverse Effects

 The blood pressure of one patient in the *F* group and two patients in the *R* group, all of whom had a preexisting comorbidity of chronic hypertension, could not be well controlled. Esmolol or nitroprusside infusions were administered to these patients in order to keep their blood pressure within the normal range. 

 By contrast, hypotension was observed in 5 patients within the *F* group. Therefore, dopamine + dobutamine + noradrenaline IV infusion was started in 2 patients, while dopamine + dobutamine iv infusion was administered in the other 3 patients to manage their hypotension. Hypotension was also observed in 5 patients within the *R* group. Two of these patients were treated with dopamine + noradrenaline infusion, and the other 3 patients were treated with dopamine + dobutamine infusion. There was no significant difference between the *F* and *R* groups statistically (*P* > 0.05). 

 Bradycardia was observed in 2 patients, both from the *R* group. This problem was managed using 0.5 mg atropine. Apart from hypotension and bradycardia, no other side effects were observed in either group. There was no recorded clinical evidence to suggest the development of delirium or tolerance to remifentanil or fentanyl.

## 4. Discussion

### 4.1. Sedation Protocols

 Where the primary goal is to achieve the earliest awakening possible a sedation protocol strategy which includes daily interruption of sedative infusions may be applied [6]. In addition, daily arousal can help to deal with the unpredictability of sedation levels and reduce the amount of sedative drug needed. Daily interruption of sedative in mechanically ventilated patients decreases the duration of mechanical ventilation and the length of stay in the ICU. It may also reduce many of the complications associated with sedatives and result in improved clinical outcomes [7]. Most interestingly, no subsequent negative long-term psychological effects are observed in patients who have been assessed on a daily basis. Moreover, long-term followup suggests that posttraumatic stress disorders are less pronounced compared to the control group [8].

 Protocols aimed at reducing the adverse effects of sedation through close monitoring and selection of agents with lower accumulative potential have proven particularly successful in critically ill patients requiring prolonged ventilatory support [9].

### 4.2. Hemodynamics

 Similar to the research of Akıncı et al. [10], in our study there were no significant differences between the *F* and *R* groups in terms of MAP, HR, or body temperature. On the other hand, the *R* group demonstrated a greater, but statistically non-significant, reduction in MAP than the *F* group (Figure 1). The similarity in the weighted mean body temperature, MAP, and HR data and the comparable incidence of patients with hemodynamic outliers in both treatment groups show that remifentanil and fentanyl both provided an acceptable degree of hemodynamic stability [10, 11]. In contrast, a study comparing bolus remifentanil with fentanyl (both at 2 *μ*g/kg) in children suggested that remifentanil lowered blood pressure, heart rate, and the rate pressure product (heart rate + systolic blood pressure) significantly more than fentanyl, after anaesthetic induction. In two of the patients, remifentanil induced bradycardia requiring atropine intervention [12].

### 4.3. Analgesics and Sedative Dosage

 Breen et al. [13] demonstrated that midazolam requirements were considerably reduced in the remifentanil group compared to fentanyl and were relatively constant throughout the treatment period. They postulated that this was a reflection of the hypnotic-agent sparing effects of remifentanil and the ease with which the infusion could be titrated to obtain optimum comfort. In our study, there were no significant differences in midazolam dosage requirement between the *F* and *R* groups (*P* > 0.05), although a nonsignificantly lower midazolam requirement in the *F* group compared to the *R* group was observed (Figure 4). In addition, the dose of remifentanil could not be increased easily because of its potential bradycardia effect. Therefore, the dosage of remifentanil was increased cautiously to obtain optimum patient comfort. A study among patients undergoing surgery for abdominal-aortic aneurysm indicated that remifentanil may provide more stable pain control than the combination of fentanyl-midazolam [14]. This proposal was not substantiated by our study.

### 4.4. Quality of Sedation and Analgesia

 In our study, midazolam-induced sedation was assessed by validated RS and SAS. Riker et al. demonstrated good validity and reliability with SAS and a good correlation between the SAS and the RS [15]. In our study also, a strong, negative correlation between RS and SAS was observed (*R* = 0.822, *P* < 0.001) (Figure 6), while there were no significant differences between the groups (*P* > 0.05). From the induction of sedation, the mean RSs for the *F* and *R* groups were between 4 and 5, while the mean SAS for both groups was observed to be between 2 and 3. These sedation scores may also indicate that patients were at times oversedated. On the other hand it should be noted that 81% of the patients in the *R* group and 75% of the patients in the *F* group were neurosurgical patients. Karabinis et al. assessed the safety and efficacy of analgesia-based sedation using remifentanil in a neurointensive care unit, and the mean SAS score was observed to be between one and three. They also did not observe any clinical differences in either pain or sedation scores [11]. In addition, they suggested that additional daily neurological assessments, such as measuring intracranial pressure (ICP) and cerebral perfusion pressure (CPP), should be undertaken in order to provide adequate sedation. 

 After analgesic therapy was initiated, a significant reduction in BPS scores was recorded compared with baseline values (*P* < 0.001). In comparison with the *F* group, the *R* group provided a significant reduction in the first 10 minutes (*P* < 0.05). Similar to our study, Akıncı et al. observed that fentanyl caused significant reduction in BPS score within 10 minutes of infusion while remifentanil achieved a similar reduction within 5 minutes (*P* < 0.05) [10]. Our study confirms findings suggesting that remifentanil has a more rapid onset of action than fentanyl [10].

 With reference to mechanical ventilation and ICU discharge time, sedation and analgesia scales play an important role in providing optimum sedoanalgesia. Botha and Mudholkar found that the introduction of a sedation scale led to a reduction in the duration of mechanical ventilation [16].

 Breen et al. demonstrated a decrease in the duration of mechanical ventilation when using remifentanil-based analgesia and sedation [13], while Amor et al. observed that the extubation time of remifentanil was shorter than that for fentanyl [17]. By contrast Akıncı et al. [10] and Karabinis et al. [11] found no difference between remifentanil and fentanyl groups concerning time to extubation. Likewise, in our study, with respect to sedation and mechanical ventilation time, there were no significant differences between groups (*P* > 0.05) (see Figure 6).

 Breen et al. [13] also observed that there was a non-significant trend towards a shorter ICU stay in the remifentanil group by one day. This is consistent with our study, as can be seen in Figure 6, where the ICU discharge time of the *R* group is approximately one day lower than that of the *F* group (*P* > 0.05). The trend towards reduced duration of ICU stay is supported by the work of Matthey et al. [18], who showed that remifentanil-based sedation significantly reduced the time on mechanical ventilation and allowed earlier discharge from the ICU than fentanyl-based sedation.

### 4.5. Organ Dysfunction

 The shorter weaning time with remifentanil is a direct consequence of its pharmacokinetic profile. Remifentanil has a rapid onset (1 minute) and offset (half life < 10 minutes) of action [19]. Its organ-independent metabolism by nonspecific blood and tissue esterases results in a pharmacokinetic profile unaffected by impaired kidney [17] and liver [20] function. It does not accumulate, even after prolonged infusion [21]. Although fentanyl has a rapid onset and a short clinical duration with single doses, accumulation and prolonged sedative effects may be observed after continuous administration. In our study, there were significant differences between the *R* and *F* groups at times of 0, 24, and 48 hours (*P* < 0.05) with respect to ALT. As shown in Table 4, ALT level was decreased in *R* group while it was increased in *F* group within the first 48 hours. In terms of BUN levels (Table 3), we found significant differences between both groups at 72 and 96 hours (*P* < 0.05). Clinically there may be little difference between the groups, but its organ-independent mode of metabolism makes remifentanil the opioid of choice for use in patients with impaired renal or hepatic functions.

### 4.6. Adverse Effects

 With respect to adverse events during the provision of adequate sedation and analgesia, no statistically significant differences could be determined [11, 13]. An in-vivo study observed no significant differences between remifentanil, fentanyl, and isoflurane in terms of motor function, anaesthetic time required for the amplitude of the third negative postsynaptic (N3) wave to disappear, or in the numbers of morphologically normal-appearing neurons seven days after reperfusion. The researchers demonstrated that high-dose fentanyl and remifentanil do not exacerbate ischaemic spinal cord injury. This may be a significant finding because many patients in ICU such as those in our study have sustained CNS damage [22].

The highest incidence of drug-related adverse effect in the *R* and *F* groups was related to hypotension. When hypotension occurred it was controlled firstly by titration of opioid dose, and then, when necessary, by the use of inotropes such as dopamine and/or dobutamine. In addition, bradycardia was observed in two patients from the *R* group, and was managed using atropine. Our findings indicate that remifentanil and fentanyl have similar adverse effect profiles.

### 4.7. Clinical Pharmacy Services in ICU

 Recent studies have reported that clinical pharmacy services can reduce hospital mortality rates [23], preventable adverse drug events [24], and medical costs [25] and improve the efficacy of treatment [26]. In addition, it has been demonstrated that the institution of a daily pharmacist-enforced intervention directed at improving sedation guideline adherence resulted in a significant decrease in the duration of mechanical ventilation in patients receiving continuous sedation [27].

## 5. Conclusion

 In conclusion, this study demonstrates that there were a small number of significant differences between remifentanil and fentanyl statistically with respect to hemodynamic parameters, analgesic and sedative requirements, sedation time, mechanical ventilation time, ICU discharge time, organ dysfunction, or adverse effects. Compared to fentanyl, remifentanil provided significantly more potent and rapid analgesia based on BPS measurements and a statistically non-significantly shorter time to discharge. On the other hand, remifentanil caused a significantly sharper fall in heart rate within the first six hours of treatment. Differences between groups in terms of BUN and ALT were also recorded. Although both fentanyl-midazolam and remifentanil-midazolam can safely provide sedation and analgesia, fine differences between the fentanyl and remifentanil should be considered when selecting a suitable regimen for individual patients. 

The employment of sedation and analgesia assessment scales such as SAS, RS, and BPS is useful to provide optimum sedation and analgesia. A strong correlation between SAS and RS was found in this study which confirms the value of these measurement criteria. These tools should therefore be utilized by the ICU team to ensure the patient is comfortable without being oversedated.

## Figures and Tables

**Table 1:** Demographic and clinical characteristics of the patients on admission to ICU.

Variables	*R* group (*n* = 16)	*F* group (*n* = 16)	*P* value
Age, yr	50.63 ± 25.24	51.88 ± 20.77	0.985
Weight, kg	65.94 ± 11.89	70.06 ± 15.12	0.539
Gender*			
M	7	10	
F	9	6	
Clinic*			
Brain surgery	13	12	
Internal	1	1	
General surgery	1	0	
Urology	0	1	
Orthopaedics	1	2	
APACHE II score	9.56 ± 3.83	11.94 ± 6.4	0.305
Pred. Mortality, %	12.3 ± 7.73	17.18 ± 14.4	0.243
GCS	11 ± 3.97	10.06 ± 4.74	0.669
MAP, mmHg	96.25 ± 17.43	86.63 ± 19.18	0.254
Heart rate, beats/min	93 ± 17.92	86.75 ± 25.17	0.305
Temperature, °C	36.5 ± 0.54	36 ± 0.47	0.080
sPO_2_, %	99 ± 0.89	97.63 ± 4.88	0.696
PaO_2_, mmHg	167.63 ± 57.38	199.3 ± 95.22	0.287
PaCO_2 _, mmHg	36.78 ± 7.96	37,14 ± 11,36	0.867
PH	7.37 ± 0.1	7.34 ± 0.12	0.381
CVP, mmHg	7.13 ± 3.86	8.38 ± 6.45	0.780
ALT, U/L	68.38 ± 65.93	37.31 ± 28.85	0.067
AST, U/L	135.93 ± 316.02	38.5 ± 22.06	0.305
BUN, mg/dL	42.75 ± 29.52	61.81 ± 79.38	0.985
SrCr, mg/dL	0.8 ± 0.3	1.35 ± 0.63	0.361

*Because the figures involved in these categories were small, statistical analysis was not deemed necessary.

**Table 2:** Mean daily RS and SAS scores during the study period.

*n* = 32	RS	SAS	*R* value
Day 1	5.70	1.55	−0.744
Day2	5.00	2.30	−0.926
Day3	4.59	2.80	−0.648
Day4	3.80	3.58	−0.357

**Table 3:** BUN and creatinine data in *F* and *R* groups at 0, 24, 48, 72, and 96 hours.

	Hours	Group *F* (*n* = 16)	Group *R* (*n* = 16)	*P* value
BUN	0	32.51 ± 6.14	37.31 ± 23.75	> 0.05
	24	30.81 ± 13.2	37.06 ± 24.45	> 0.05
	48	28.62 ± 14.01	36.56 ± 24.23	> 0.05
	72	29.56 ± 16.10	31.25 ± 18.90	< 0.05
	96	28.80 ± 16.24	26.38 ± 17.74	< 0.05

SCr	0	2.28 ± 4.47	0.80 ± 0.30	> 0.05
	24	2.05 ± 4.16	0.78 ± 0.35	> 0.05
	48	1.61 ± 2.47	0.75 ± 0.34	> 0.05
	72	1.54 ± 2.42	0.75 ± 0.33	> 0.05
	96	1.52 ± 2.41	0.73 ± 0.31	> 0.05

**Table 4:** Liver enzyme data in *F* and *R* groups at 0, 24, 48, 72, and 96 hours.

	Hours	Group *F* (*n* = 16)	Group *R* (*n* = 16)	*P* value
ALT	0	37.31 ± 31.25	68.38 ± 65.93	< 0.05
	24	41.06 ± 28.25	65.81 ± 62.52	< 0.05
	48	42.53 ± 25.15	48.80 ± 36.90	< 0.05
	72	39.13 ± 19.20	43.25 ± 30.70	> 0.05
	96	34.44 ± 12.32	42.25 ± 26.72	> 0.05

AST	0	38.50 ± 22.06	45.38 ± 28.23	> 0.05
	24	40.01 ± 22.75	44.06 ± 23.64	> 0.05
	48	41.44 ± 17.36	38.50 ± 19.39	> 0.05
	72	42.81 ± 21.65	39.01 ± 18.48	> 0.05
	96	42.75 ± 19.67	46.38 ± 31.09	> 0.05
